# Retraction: Laparoscopic vs. open pancreaticoduodenectomy after learning curve: A systematic review and meta-analysis of single-center studies

**DOI:** 10.3389/fsurg.2022.988654

**Published:** 2022-07-26

**Authors:** Frontiers Editorial Office

**Affiliations:** Frontiers Media SA, Lausanne, Switzerland

**Keywords:** pancreatic cancer, pancreaticoduodenectomy, laparoscopic, whipple, meta-analysis

## A Retraction of the Systematic Review Article

Laparoscopic vs. Open Pancreaticoduodenectomy After Learning Curve: A Systematic Review and Meta-Analysis of Single-Center Studies

By Feng Q, Xin Z, Qiu J and Xu M (2021). Front. Surg. 8:715083. doi: 10.3389/fsurg.2021.715083

The journal and Chief Editors retract the 10 September 2021 article cited above.

“*Following publication, the cited article was found to be substantially similar to a previously published study by Feng et al. in Gland Surgery (2021;10(5):1655–1668; doi: 10.21037/gs-20-916). Our investigation, conducted in accordance with Frontiers’ policies, concluded that this similarity was unacceptably high, and the Publisher is retracting the article*.

*This retraction was approved by the Chief Editors of Frontiers in Surgery and the Chief Executive Editor of Frontiers. The authors have agreed to the retraction.”*

